# Unusual Structures of Interpolyelectrolyte Complexes: Vesicles and Perforated Vesicles

**DOI:** 10.3390/polym12040871

**Published:** 2020-04-10

**Authors:** A. A. Glagoleva, D. E. Larin, V. V. Vasilevskaya

**Affiliations:** A. N. Nesmeyanov Institute of Organoelement Compounds, Russian Academy of Sciences, Moscow 119991, Russia; starostina@polly.phys.msu.ru (A.A.G.); larin@polly.phys.msu.ru (D.E.L.)

**Keywords:** interpolyelectrolyte complexes, self-organization, vesicles

## Abstract

By means of computer simulation and analytical theory, we first demonstrated that the interpolyelectrolyte complexes in dilute solution can spontaneously form hollow spherical particles with thin continuous shells (vesicles) or with porous shells (perforated vesicles) if the polyions forming the complex differ in their affinity for the solvent. The solvent was considered good for the nonionic groups of one macroion and its quality was varied for the nonionic groups of the other macroion. It was found that if the electrostatic interactions are weak compared to the attraction induced by the hydrophobicity of the monomer units, the complex in poor solvent tends to form “dense core–loose shell” structures of different shapes. The strong electrostatic interactions favor the formation of the layered, the hollow, and the filled structured morphologies with the strongly segregated macroions. Vesicles with perforated walls were distinguished as the intermediate between the vesicular and the structured solid morphologies. The order parameter based on the spherical harmonics expansion was introduced to calculate the pore distribution in the perforated vesicles depending on the solvent quality. The conditions of the core–shell and hollow vesicular-like morphologies formation were determined theoretically via the calculations of their free energy. The results of the simulation and theoretical approaches are in good agreement.

## 1. Introduction

Interpolyelectrolyte complexes (IPECs) are formed in solutions by oppositely charged polyions. IPECs play an important role in nature; for example, polyelectrolyte complexation occurs in chromatine fiber, formed by DNA and histones [1], and within cells in the interaction between DNA and enzyme proteins [2]. Water-soluble IPECs are promising for various biomedical applications including drug encapsulation and targeted delivery, gene therapy, gene transfection [3,4,5,6,7,8,9], as well as for applications in water treatment, agriculture, nanofiltration, etc. [10,11].

The formation of IPECs is driven by electrostatic interactions between the oppositely charged groups, as well as entropy change upon the release of counterions, which were bound to the free polyions [12,13,14,15]. The morphology and properties of IPECs are, to a large extent, determined by the density of charges within the macromolecules and the ratio between positive and negative charges [16,17,18]. If the charges of one sign prevail, the complexes are called nonstoichiometric. They form micelles with the charged surface and are water-soluble due to the electrostatic interaction between water and charged monomer units. Solutions of oppositely charged polyelectrolytes, which carry an equal amount of positive and negative charges (stoichiometric case), have zero net charge and reveal macroscopic separation into polymer-rich and dilute phases [7,18,19,20,21].

However, in stoichiometric IPECs, phase separation can be avoided if at least one of the polyelectrolytes contains nonionic hydrophilic groups. That is the case in block-ionomer complexes, forming core–shell structures with the polyelectrolyte complex core stabilized by the hydrophilic shell of the nonionic block [22,23]. In addition, the soluble IPECs of the core–shell structure can be obtained when the hydrophilic groups are distributed evenly along the linear polyelectrolyte chains: In the IPECs studied in [24,25], the uncharged groups are hydrophobic in polycations and hydrophilic in polyanions. The outer part of the resulting complex comprises hydrophilic groups, tending to be exposed into the solvent and shielding the complex from aggregation, while the inner part consists of both hydrophilic and hydrophobic units. The theory of such IPECs is proposed in [25]. Within the framework of this theory, the thickness of the external layer of the IPEC is determined by the two opposite effects: The energetic gain from hydrophilic groups immersed into the solvent and the electrostatic attraction between the excess of negative and positive groups in the core and shell of the IPEC, respectively. 

It can be assumed that the core of the complexes can possess some ordered distribution of the groups as a result of the interaction between the charged groups, as well as the interaction of hydrophobic and hydrophilic units with the solvent. We have investigated the morphology of such IPECs by means of computer simulation for the case of strong electrostatic attraction between the oppositely charged chains and gradual decrease in the solvent quality for one of the chains, while the solvent quality for the other chain remained good [26]. It was demonstrated that the complexes acquired a near-spherical shape with the solvophilic outer shell. Upon worsening of the solvent, the monomer units within the inner part of the complex rearranged so that one could distinguish three morphology types depending on the solvent quality: The particles with loosely packed monomer units in the core surrounded by the solvophilic shell, the hollow “vesicle-like” particles, and the densely structured particles with strongly segregated chains. The transition between the vesicle and the structured dense particle proceeds via the formation of the hollow structures with the perforated shells [26].

In the studied IPEC, the macromolecules with different affinities for the solvent are bound together by strong electrostatic interactions as if they were parts of a single chain [26]. Therefore, the complex has an amphiphilic nature and resembles an amphiphilic homopolymer [27,28,29], which has both solvophilic and solvophobic groups within each monomer unit. It is known that in solution, amphiphilic homopolymers can form a number of morphologies with the solvophilic shell, including hollow vesicle-like structures [27,28,29,30,31,32,33,34]. 

The nanoparticles with a hollow morphology are especially promising as nanocarriers. It was shown [35] that encapsulation of peptides into hollow nanoparticles instead of solid ones enhances release and preserves the bioactivity of peptides due to the decrease in the interaction with the polymer matrix. Amphiphilic molecules are widely used to create hollow capsules. Those are lipids and various systems with enhanced stability and control: The complexes of lipids with other molecules, synthetic lipid-like materials, block-copolymer polymersomes [36,37,38,39]. Application of amphiphilic homopolymers [28,29], where the amphiphiles are joined into one chain, instead of the smaller molecules, suggests more straightforward ways to construct vesicles and control their morphology. In addition to hydrophobic interactions, electrostatic interactions make a significant contribution to the self-organization and stability of hollow structures [40,41,42]. Preparing hollow IPEC microcapsules nowadays involves complicated techniques such as multilayering [43,44] and interface encapsulation [45,46]. We assume that the utilization of electrostatic interactions in IPECs as a tool for effectively “binding” the chains with a different affinity for the solvent and, thus, the introduction of amphiphilicity opens the way for the development of novel methods to obtain stable and controllable nanocapsules.

The aim of the present study is to investigate the shape and distribution of monomer units in stoichiometric interpolyelectrolyte complexes consisting of two chains having different affinities for the solvent, depending on the length of the chains, solvent quality, and the Bjerrum length; namely, to obtain the conditions for the emergence of various morphologies, indicate the area of the hollow structures formation, and quantitatively describe the shapes of these hollow structures.

## 2. Computer Simulation 

### 2.1. Model and Simulation Technique

**Figure 1:**
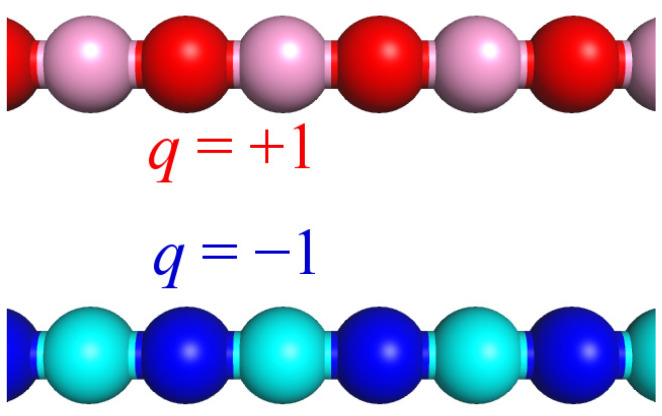
Schematic representation of the studied macromolecules. In all the snapshots, non-charged and charged groups of the hydrophobic chain (polycation) are depicted by light-red and dark-red beads, respectively, while non-charged and charged groups of the hydrophilic chain (polyanion) are shown by light-blue and dark-blue beads, respectively.

The system under study consisted of two polymer chains of similar length *N* and a similar degree of ionization *f* = ½. Each chain bore *N*/2 charged and *N*/2 uncharged monomer units distributed in an alternating way (Figure 1). The first chain was charged positively while the second chain was charged negatively. The magnitude of the charges was *q* = +1 and *q* = −1, respectively. The length of the macromolecules was varied from *N* = 512 to 2048. The complex was immersed into the solvent, which was considered good for the polyanion and whose quality was varied for the polycation.

Computer simulations of the temporal evolution of the system were carried out using the Langevin molecular dynamics technique applying the LAMMPS software package [47].

The bonds between the monomer units were described by the rigid spring potential:

(1) $$E_{bond}=K{\left(r_{ij}-b\right)}^{2}$$

where b=1 is a bond length, and K=10,000 is a constant with a numerical value sufficient to ensure that the bond length deviation does not exceed 1%.

The excluded volume interactions between the non-bonded monomer units were introduced via the repulsive part of the Lennard–Jones potential:

(2) $$E_{LJ}(r_{ij})=\left\{ \begin{array}{l} 4\varepsilon_{LJ}\left[{\left(\frac{\sigma }{r_{ij}}\right)}^{12}-{\left(\frac{\sigma }{r_{ij}}\right)}^{6}+\frac{1}{4}\right]\\ 0,\;r_{ij}>r_{c} \end{array}\right.,r_{ij}\leq r_{c}$$

where $r_{ij}$ is the distance between *i*-th and *j*-th units, $r_{c}=\sqrt[{6}]{2}\sigma$ is the cutoff distance of the potential, $\varepsilon_{LJ}$ is a parameter controlling the energy scale, and σ determines the length scale. We put $\varepsilon_{LJ}=\sigma =1$ so all of the results are presented in terms of the parameters $\varepsilon_{LJ}$ and σ.

The solvent was represented implicitly by a dielectric continuum. Hydrophobic–hydrophilic intrachain interactions, induced by the solvent, were implicitly accounted for by the Yukawa-type potential:

(3) $$E_{Y(\alpha \beta )}(r_{ij})=\left\{ \begin{array}{l} \frac{\varepsilon_{\alpha \beta }\sigma }{r_{ij}}{\left(1-{\left(\frac{r_{ij}}{r_{cY}}\right)}^{2}\right)}^{2},r_{ij}\leq r_{cY}\\ 0,r_{ij}>r_{cY} \end{array}\right.$$

where $r_{cY}=4\sigma$ is the cutoff distance and $\varepsilon_{\alpha \beta }=(\varepsilon_{HH},\varepsilon_{PP},\varepsilon_{HP})$ is the energetic parameter of pair H–H, P–P, and H–P interactions, H and P being monomer units of the first (hydrophobic) and second (hydrophilic) chains, respectively. The parameter $\varepsilon_{HH}$ had negative values ($\varepsilon_{HH}<0$), demonstrating the effective attraction between the units of the hydrophobic chain due to its low affinity for the solvent. To simulate the good solvent conditions for the hydrophilic chain, the values of $\varepsilon_{HP}$ and $\varepsilon_{PP}$ were set to zero, i.e., only excluded-volume interactions (Equation (2)) were acting between H–P and P–P pairs.

Electrostatic interactions between the charged units were presented via the Coulomb potential:

(4) $$E_{El}(r_{ij})=k_{B}Tl_{B}\frac{q_{i}q_{j}}{r_{ij}}$$

where $k_{B}T=1$, $r_{ij}$ is the distance between *i*-th and *j*-th units, and $l_{B}=\frac{e^{2}}{4\pi \varepsilon_{0}\varepsilon k_{B}T}$ is the Bjerrum length (*e* is an elementary charge, ε is the dielectric permittivity of the medium, ε_0_ is vacuum permittivity). For charged monomer units in the first chain, $q_{i}=+1$, and for those in the second chain, $q_{i}=-1$. Electrostatic interactions were calculated using the Ewald summation algorithm [48]. The Bjerrum length was varied from *l_B_* = 0.25 to 10. Bjerrum length is the distance at which the electrostatic energies of the two charged units become equal to *k_B_T*. Therefore, the electrostatic energy of the two charge interactions can be considered small with respect to the thermal energy if the distance between them is large enough compared to the Bjerrum length. In the article, for convenience, we refer to small and large values of *l_B_* as “weak” and “strong” electrostatic interactions, respectively. 

We consider very dilute solutions of oppositely charged polyelectrolytes. When a compact stoichiometric complex is formed, the counterions of both polyions are released into the whole solution volume due to their high translational entropy [12,13]. Therefore, most of the counterions were assumed to be located far from the complex and their influence was neglected.

The solution temperature *T* was kept constant using a Langevin thermostat. The equations of motion were augmented by the Langevin uncorrelated noise term $F_{r}\sim \sqrt{\frac{2k_{B}Tm_{b}}{Ddt}}$ and drag force term $F_{f}=-\frac{m_{b}}{D}v$. Here, $m_{b}=1$ is the mass of each group, v is its velocity, $dt=0.005\sigma \sqrt{\frac{m_{b}}{\varepsilon_{LJ}}}$ is the integration step, and *D* = 100 is the damping coefficient, indicating that the temperature is relaxed within 100 time steps.

The macromolecules were placed into a large cubic simulation cell of edge *L* = 1200 or 2400 (depending on chain length) with periodic boundary conditions. Relaxation was carried out at $\varepsilon_{HH}=\varepsilon_{PP}=\varepsilon_{HP}=0$ for 4 × 10^6^ time steps. Then, the parameter $\varepsilon_{HH}$ of the interaction between the first chain groups was switched on and changed gradually from $\varepsilon_{HH}=0$ to $\varepsilon_{HH}=-2.0$ with step $\Delta \varepsilon_{HH}=-0.1$, performing an equilibration run of 2 × 10^6^ and production run of 2 × 10^6^ time steps at each stage.

### 2.2. Results and Discussion

**Figure 2:**
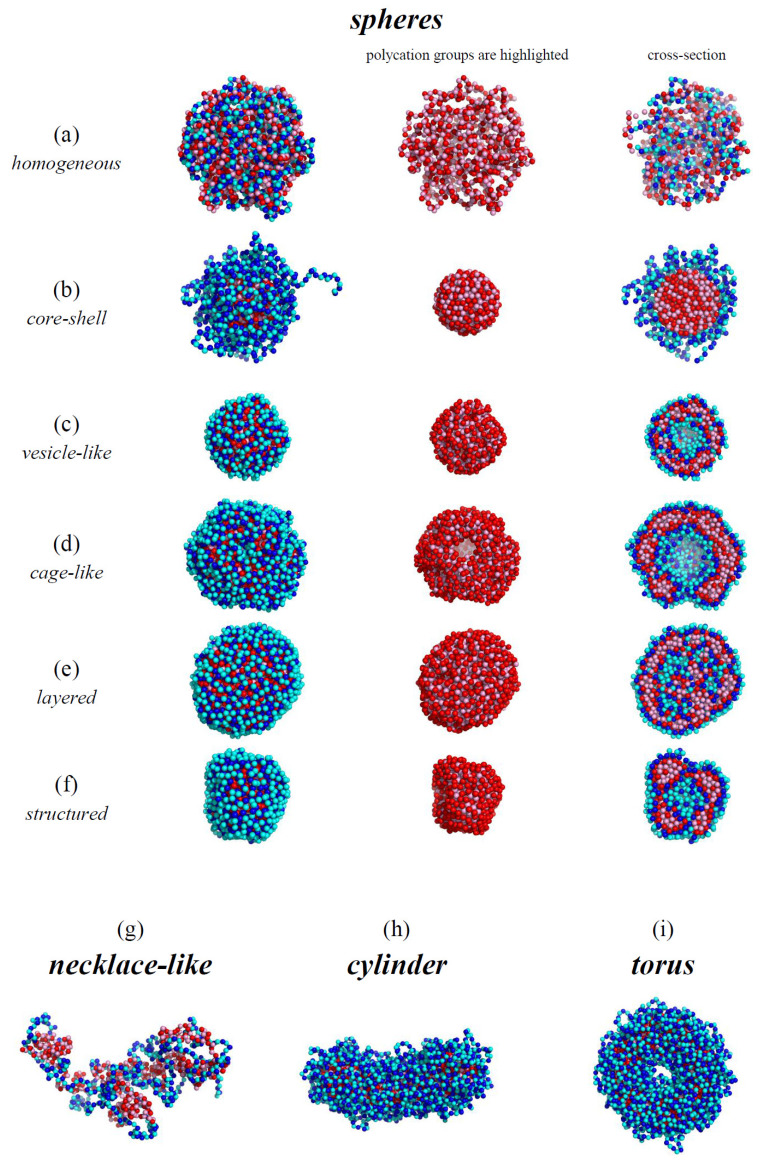
The snapshots of the typical morphologies formed by the complex: *l_B_* = 4, *N* = 1024, $\varepsilon_{HH}=0$ (**a**); *l_B_* = 0.25, *N* = 1024, $\varepsilon_{HH}=-2.0$ (**b**); *l_B_* = 10, *N* = 1024, $\varepsilon_{HH}=-2.0$ (**c**); *l_B_* = 4, *N* = 2048, $\varepsilon_{HH}=-2.0$ (**d**); *l_B_* = 10, *N* = 2048, $\varepsilon_{HH}=-2.0$ (**e**); *l_B_* = 6, *N* = 1024, $\varepsilon_{HH}=-3.0$ (**f**); *l_B_* = 0.25, *N* = 512, $\varepsilon_{HH}=-0.3$ (**g**); *l_B_* = 0.5, *N* = 2048, $\varepsilon_{HH}=-2.0$ (**h**); *l_B_* = 1, *N* = 2048, $\varepsilon_{HH}=-2.0$ (**i**).

The characteristic types of morphologies observed in our calculations are shown in Figure 2. There are spherical (Figure 2a–f), necklace-like (Figure 2g), cylindrical (Figure 2h), and toroidal micelles (Figure 2i). The spherical micelles either have a homogeneously filled (Figure 2a,b), hollow (Figure 2c,d), layered (Figure 2e), or structured interior (Figure 2f). The hollow structures can have a vesicular-like shape with continuous walls (Figure 2c) or a cage-like shape, i.e., porous walls (Figure 2d). The complexes in good solvent (Figure 2a) possess a structure with homogeneously intermixed chains. It is seen that the outer layer of all the other structures (Figure 2b–i) and the inner layer of the hollow morphologies (Figure 2c,d) are built by the hydrophilic chain. The types of the conformations are similar to those we have observed earlier formed by amphiphilic homopolymers [32]. As we have demonstrated [26], the complexes of the polyions, which have different affinities for the solvent, bear resemblance to the amphiphilic macromolecules as the hydrophilic and hydrophobic chains are bound in them by strong electrostatic interactions.

The types of structures formed by the complex depend on the solvent quality, the length of the chains, and the strength of the electrostatic interactions expressed by the value of Bjerrum length *l_B_*. We have calculated several quantities determining the structure of the complexes in order to systematize the results and present them in the form of morphological diagrams.

To distinguish between the spherical, cylindrical, and toroidal morphologies, the shape factors *k*_1_ and *k*_2_ were calculated. These values are defined as the ratios between the principal moments of the squared gyration radius ($g_{1}\leq g_{2}\leq g_{3}$):

(5) $$k_{1}=\frac{g_{1}+g_{2}}{g_{2}+g_{3}},\;k_{2}=\frac{g_{1}+g_{3}}{g_{2}+g_{3}}$$

For the perfect sphere $k_{1}\to 1$, $k_{2}\to 1$; for the infinitely thin disk $k_{1}\to 0.5$, $k_{2}\to 0.5$; for the infinitely long cylinder $k_{1}\to 0$, $k_{2}\to 1$.

**Figure 3:**
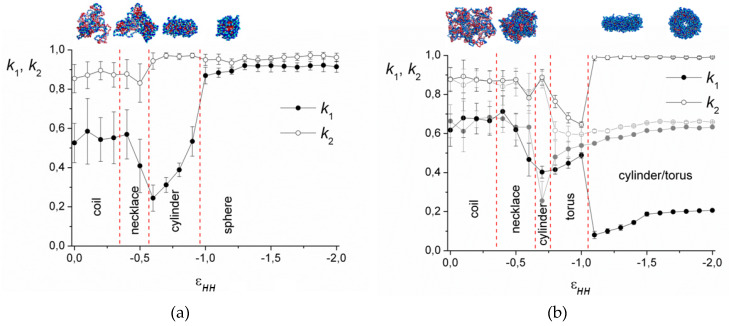
Shape factors and snapshots of the complexes with *N* = 512 (**a**) and *N* = 2048 (**b**). *l_B_* = 0.5.

The dependencies of the shape factors on the solvent quality parameter $\varepsilon_{HH}$ for the case of low electrostatic interactions (*l_B_* = 0.5) are demonstrated in Figure 3. As is seen, in good solvent, their values are *k*_1_ ≈ 0.55, *k*_2_ ≈ 0.85 for short chains (Figure 3a) and *k*_1_ ≈ 0.65, *k*_2_ ≈ 0.90 for long chains (Figure 3b) and strongly fluctuate. With the decrease in the solvent quality, the value of *k*_1_ diminishes and *k*_2_ remains the same, which indicates elongation of the complex. A further decrease in solvent quality for the short chains leads to the growth of both *k*_1_ and *k*_2_ up to *k*_1_ ≈ *k*_2_ ≈ 0.9, corresponding to the spherical micelles. The long macromolecules demonstrate coexistence of the toroidal and cylindrical morphologies (Figure 3b).

**Figure 4:**
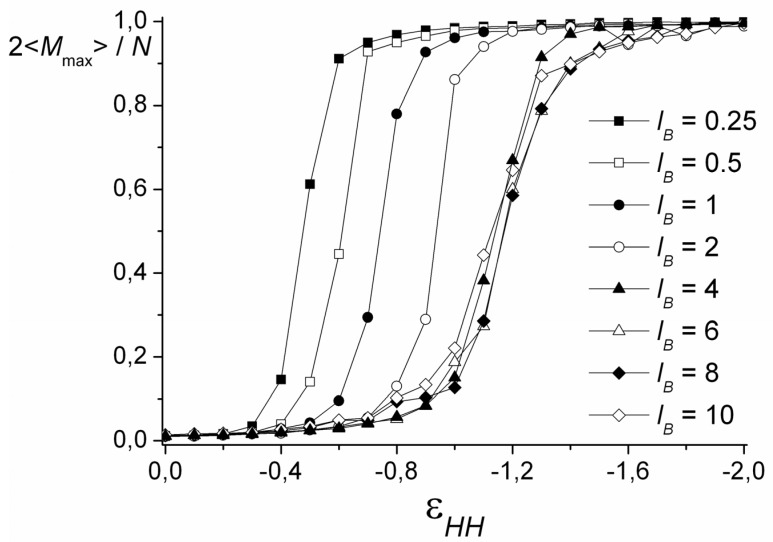
The size of the maximal cluster formed by nonionic groups of the polycation depending on the solvent quality. *N* = 1024.

On the snapshots (Figure 3), it is seen that the coil transforms into the cylinder via the stage of the necklace formation. The beads in the necklace are strongly entangled and, visually, they are the areas with the dense cores of polycation groups and the shells of polyanion groups. The necklace region was found by calculation of <*M_max_*>, the size of the maximal cluster formed by nonionic groups of the polycation. The cluster is a group of units, where the distance *r_ij_* between each pair does not exceed the critical value $r_{c}=1.4\sigma$. Figure 4 shows the dependencies of 2<*M_max_*>/*N* on the values of $\varepsilon_{HH}$. The chain length does not significantly affect the shape of the curves. In good solvent, the clusters of maximal size are very small and each of them comprises ~2% of all nonionic groups of the polycation. Upon worsening of the solvent, the value of <*M_max_*> increases abruptly within the narrow region of $\varepsilon_{HH}$ and reaches the plateau 2<*M_max_*>/*N* → 1. The point, where most of the units join the cluster of maximal size (2<*M_max_*>/*N* → 1), shifts to the area of the poor solvent as the *l_B_* value grows until the curves converge at *l_B_* ≥ 4. The morphology belongs to the necklace region if the point on the aggregation number dependency falls into the range of the abrupt 2<*M_max_*>/*N* rise, and the shape is intermediate between the sphere and the cylinder according to *k*_1_, *k*_2_ values.

**Figure 5:**
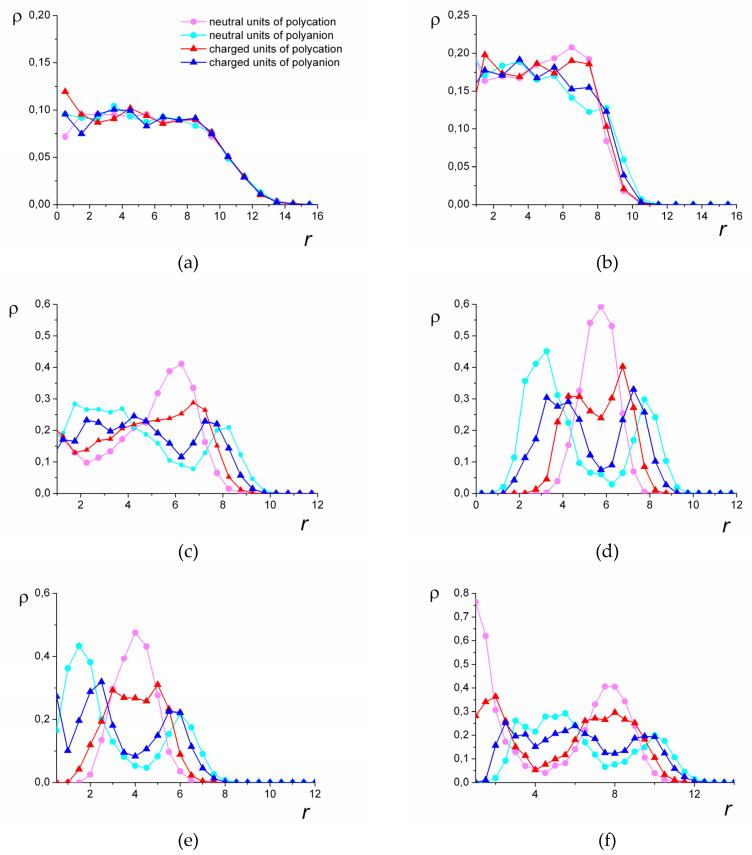
The spatial distribution of the groups: *N* = 1024, *l_B_* = 6, $\varepsilon_{HH}=0$ (**a**), $\varepsilon_{HH}=-0.9$ (**b**), $\varepsilon_{HH}=-1.5$ (**c**), $\varepsilon_{HH}=-1.8$ (**d**), *N* = 512, *l_B_* = 6, $\varepsilon_{HH}=-1.4$ (**e**), *N* = 2048, *l_B_* = 4, $\varepsilon_{HH}=-1.5$ (**f**). *r* is the distance from the center of mass. The color of the curves corresponds to the types of the groups.

Calculations of the shape factors *k*_1_ and *k*_2_ reveal that with the increase in electrostatic interactions, the shape of the complex remains spherical in a wide range of solvent qualities. In the spherical morphologies, the distribution of the charged and non-charged groups of both polyions relative to the center of mass was calculated (Figure 5).

In good solvent, the distribution is uniform for all the types of groups (Figure 5a). The worsening of the solvent quality leads to the redistribution of the monomer units. The fact that aside from the strong attraction between the groups of different polyions, the groups of the polycations prefer to be surrounded by the units of the same chain, leads to the emergence of layers within the complex. 

Figure 5b–d show how the structure evolves with the worsening of the solvent quality for the polycation groups. The layering begins at the periphery of the complex, where the shell with a predominance of the hydrophilic groups emerges (Figure 5b). Beneath this shell, there is a layer where the hydrophobic groups prevail. In the central part of the complex, the units are distributed uniformly. With the increase in the hydrophobic interactions, segregation within the central part of the complex takes place (Figure 5c). There, the local concentration of the groups of the hydrophilic chain rises, and the local concentration of the groups of the hydrophobic chain decreases. The radius of that inner core is approximately equal to the thickness of the hydrophobic layer.

As a result of the further worsening of the solvent quality, the density of the polycation groups falls to zero in the central part of the complex. Depending on the length *N* of the chains, the central part can be hollow (*N* = 1024, Figure 5d) or can be filled with the groups of the hydrophilic chain (*N* = 512, Figure 5e). The complexes of the longest chains studied (*N* = 2048) with the decrease in the solvent quality transfer into the hollow morphology via the stage where they have the additional layer. In that case, inside the central hydrophilic region, there is a core enriched by the hydrophobic groups (Figure 5f). This layered morphology is called the “onion-like” structure.

In poor solvent (the highest attraction $\left|\varepsilon_{HH}\right|$ between the groups of the hydrophobic chain), the hollow morphologies turn into solid micelles with distinctly segregated units of different polyions (Figure 1f). In those micelles, the space previously occupied by the cavity is filled with the units of the hydrophilic chain.

Besides the vesicle-like structures with a continuous shell of unaltered thickness, a number of other hollow morphologies were observed. These morphologies occupy the area between the vesicles and the so-called structured solid particles. The hollow particles with porous shells are reported in numerous studies of amphiphilic systems and referred to as cage-like micelles, stomatosomes, or perforated vesicles [49,50,51,52,53].

**Figure 6:**
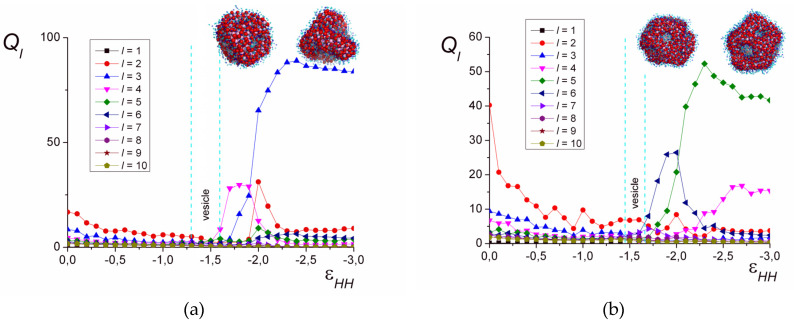
The order parameter *Q_l_* depending on $\varepsilon_{HH}$. *l_B_* = 4, *N* = 1024 (**a**), *N* = 2048 (**b**).

Figure 6 shows snapshots of the vesicles with perforated shells. As is seen, the shells of the particles undergo shape transitions. The size of the cavities inside the perforated vesicles reduces with the worsening of the solvent [26] and their shapes are maintained for the structured solid particles, which emerge in poor solvent.

We introduce the order parameter *Q_l_* to describe the shape of the hollow particles with perforated shells and the structured solid particles. First, we calculate the number w(θ,φ) of groups of polycations within the region with spherical coordinates [θ,φ ;θ+Δθ,φ+Δφ], Δθ=Δφ=π/18:

(6a) $$w(\theta ,\varphi )=\sum_{i=1}^{N}\delta (\cos\theta -\cos\theta_{i})\delta (\varphi -\varphi_{i})$$

The parameter *Q_l_* is calculated based on the spherical harmonics expansion of w(θ,φ):

(6b) $$w(\theta ,\varphi )=\sum_{l=0}^{\infty }\sum_{m=-l}^{l}w_{l}^{m}Y_{l}^{m}(\theta ,\varphi )$$

where $Y_{l}^{m}(\theta ,\varphi )$ are spherical harmonics:

(6c) $$Y_{l}^{m}(\theta ,\varphi )=\sqrt{\frac{2l+1}{4\pi }\frac{(l-m)!}{(l+m)!}}P_{l}^{m}(\cos\theta )\exp(im\varphi )$$

$P_{l}^{m}(\cos\theta )$ are Legendre polynomials, $w_{l}^{m}$ is the expansion coefficient:

(6d) $$w_{l}^{m}=\sum_{i=1}^{N}Y_{l}^{*m}(\theta_{i},\varphi_{i})$$

*i* is the number of a monomer unit within the polycation chain.

According to the addition theorem of spherical harmonics [54]:

(7) $$P_{l}(\cos\gamma_{ij})=\frac{4\pi }{2l+1}\sum_{m=-l}^{l}Y_{l}^{*m}(\theta_{i},\varphi_{i})Y_{l}^{m}(\theta_{j},\varphi_{j})$$

Here, $\gamma_{ij}$ is the angle between the vectors $\left(\theta_{i},\varphi_{i}\right)$ and $\left(\theta_{j},\varphi_{j}\right)$: $\cos\gamma_{ij}=\cos\theta_{i}\cos\theta_{j}+\sin\theta_{i}\sin\theta_{j}\cos(\varphi_{i}-\varphi_{j})$.

The order parameters $Q_{l}$, which are invariant under rotation of the coordinate system, are defined as:

(8) $$Q_{l}=\frac{1}{2l+1}\frac{4\pi }{N}\sum_{m=-l}^{l}w_{l}^{m}w_{l}^{*m}=\frac{1}{N}\sum_{i=1}^{N}\sum_{j=1}^{N}P_{l}(\cos\gamma_{ij})$$

We calculate the parameters up to *l* = 10.

Spherical harmonics expansion is applicable to measure the symmetry of noncrystallographic clusters (e.g., in the models of glasses and intermetallic compounds [55,56,57,58]) and estimate the lateral ordering of the micelles surface [59,60,61]. 

In accordance with our definition of *Q_l_*, a spherical shell with a homogeneous distribution of monomer units should have approximately equal values of the order parameter *Q_l_* for different *l*. The value of *Q*_2_ corresponds to the elongation of the micelle. The other *Q_l_* are related to the preferred density distribution in the vertices of some polyhedron, whereas the pores in the vesicular walls are located on the faces of that polyhedron. *Q_l_* can be compared to the order parameters for standard geometric shapes, which can be easily calculated. For example, the shapes with cubic symmetry have non-zero values of *Q*_2_, *Q*_4_, and *Q*_8_, and icosahedral symmetry gives non-zero values of *Q*_6_ and *Q*_10_. A tetrahedron has the following non-zero *Q_l_* (in decreasing order of magnitude): *Q*_3_, *Q*_10_, *Q*_7_, *Q*_9_, and *Q*_4_. The appearance of additional non-zero *Q_l_* values is useful to distinguish the transitions between symmetric and slightly distorted structures.

Figure 6 demonstrates the dependencies of *Q_l_* on the solvent quality parameter $\varepsilon_{HH}$ for different chain lengths. In good solvent, the shape of the complex is very fluid and varies with time from spherical to oblate, which leads to elevated *Q*_2_ values.

In Figure 6a (*l_B_* = 4, *N* = 1024), it is seen that the magnitudes of *Q_l_* for various *l* are comparable to each other and have low values in the range of $\varepsilon_{HH}$ where either spheres or the vesicles with a solid, continuous shell are formed. With the decrease in solvent quality ($\varepsilon_{HH}\leq -1.7$), some of the *Q_l_* values differ significantly in magnitude from the others. In the range of $-1.9\leq \varepsilon_{HH}\leq -1.7$, the values of *Q*_3_ and *Q*_4_ prevail. First ($\varepsilon_{HH}=-1.7;\;\varepsilon_{HH}=-1.8$), *Q*_4_ has the highest value; then ($\varepsilon_{HH}=-1.9$), the magnitudes of *Q*_3_ and *Q*_4_ become comparable. The further decrease in the solvent quality leads to the decline in *Q*_4_, significant growth in *Q*_3_, and moderate growth in *Q*_2_. That corresponds to the transition of the perforated vesicle from the symmetry most close to the cubic one, with the gradual distortion in a narrow solvent quality range. The number of pores in the shell decreases and the resulting shape of the hollow particle has three pores arranged in the vertices of the triangle, and the value of *Q*_3_ significantly exceeds all the other *Q_l_*. This symmetry is maintained both for the hollow particles ($\varepsilon_{HH}=-2.2;\;\varepsilon_{HH}=-2.3$) and the structured solid particles ($\varepsilon_{HH}\leq -2.4$).

For the long chains (*N* = 2048), the values of *Q_l_* remain small in the area of isotropic vesicles with a continuous shell ($-1.6\leq \varepsilon_{HH}\leq -1.5$), except for *Q*_2_, corresponding to the elongation of the vesicle (Figure 6b). However, the worsening of the solvent quality reveals the growth of *Q*_6_ ($-2.0\leq \varepsilon_{HH}\leq -1.7$): There, the perforated capsule has the shape of a regular polyhedron with 20 vertices and 12 faces (dodecahedron). At $\varepsilon_{HH}=-2.0$, *Q*_5_ has a noticeable value along with *Q*_6_, which corresponds to the distorted dodecahedron. The further decrease in solvent quality is accompanied by the growth in *Q*_5_ and the decline in *Q*_6_ and leads to the transition to the region, where *Q*_5_ has the largest value, the value of *Q*_4_ is smaller but also prominent, and *Q*_6_ is very low. There, the pores in the perforated vesicle shell are arranged so that they are located within the square and pentagonal faces of the polyhedron with 14 vertices and 9 faces.

Thus, for the chains of different length, the order parameter *Q_l_* reveals the rearrangement and reduction of the amount of pores in the shell of the perforated vesicles. The symmetry the shell has when the size of the inner cavity is minimal is maintained in the poor solvent, where the cavity completely disappears.

**Figure 7:**
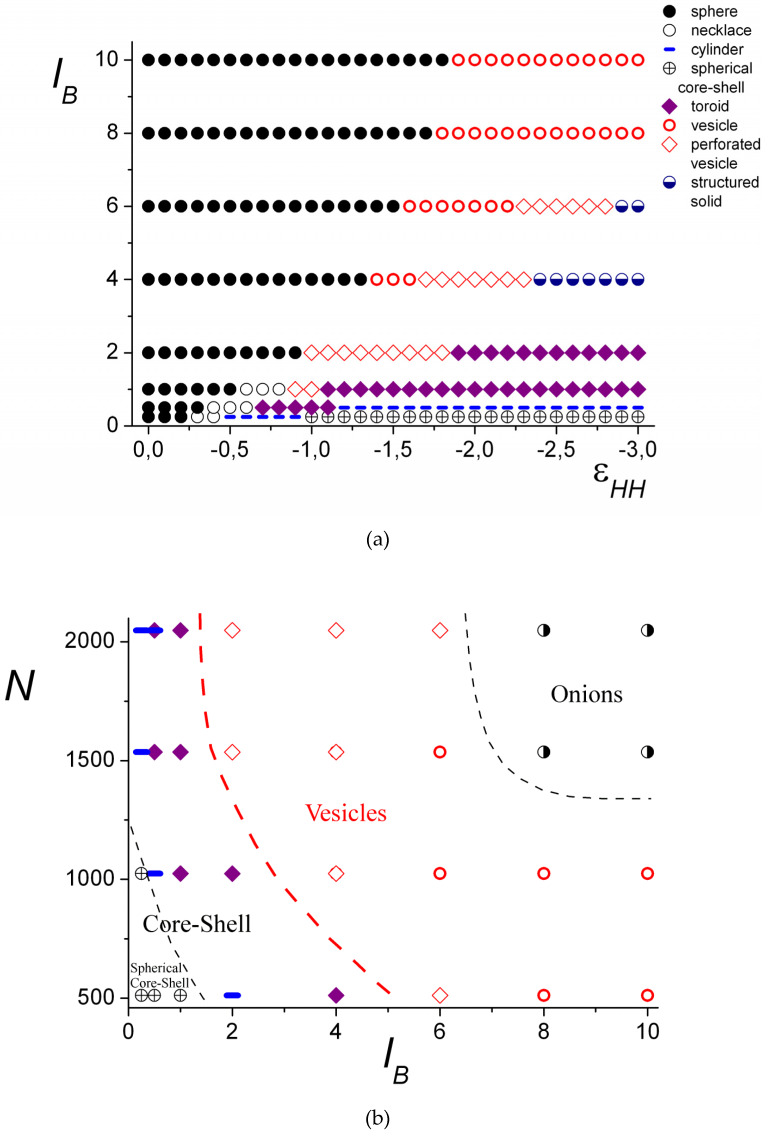
The morphological diagrams “solvent quality vs. Bjerrum length,” *N* = 1024 (**a**) and “Bjerrum length vs. chain length,” $\varepsilon_{HH}$ = − 2.0 (**b**).

Morphological diagrams in coordinates “solvent quality vs. Bjerrum length” (*N* = 1024) and “Bjerrum length vs. chain length,” summarizing the obtained results, are presented in Figure 7. Figure 7b is plotted for the case of poor solvent quality for the hydrophobic chain ($\varepsilon_{HH}=-2.0$).

In good solvent ($-0.2\leq \varepsilon_{HH}\leq 0$), all the complexes have a spherical shape. The spherical morphologies with the filled interior are denoted in the diagram by black circles. For the higher values of *l_B_*, the inner core remains filled in the wider range of $\varepsilon_{HH}$ (Figure 7a). In the diagram, the black circles designate the structures with a homogeneous core (Figure 5a,b) and those having the layers, where the difference in the concentration of the groups of different polyions is small (Figure 5c).

Upon worsening of the solvent quality, if the electrostatic interaction is weak, i.e., the values of *l_B_* are low, shorter macromolecules form spherical complexes with the dense core and loose shell (Figure 7, circles with a cross), and longer chains form cylindrical (Figure 7, blue dashes) and toroidal (Figure 7, purple rhombi) morphologies. The increase in the chain lengths leads to the narrowing of the torus region.

If the electrostatic interactions are strong, the region of the spherical conformations, including filled and hollow ones, enlarges. This region spreads from the good solvent area and gradually broadens upon *l_B_* growth until it occupies the whole studied solvent quality range (Figure 7a). In this case, the hollow structures emerge in the poor solvent. First, upon the worsening of the solvent quality, the vesicle-like morphologies are formed (Figure 7, red circles). With the further decrease in the solvent quality, they transform into the hollow structures with the porous shell (Figure 7, red rhombi), and finally into the structured solid particles, where the groups of the polyanion and the polycation are segregated. That can be either a morphology that maintains the distribution of the pores like in the perforated vesicles (Figure 7, half-shaded blue circles), or a torus.

The shorter chains have a relatively narrow area of the vesicles on the diagram (Figure 7b). There (*N* = 512), the vesicles and perforated vesicles have extremely small cavities inside with a size comparable to that of the monomer unit. Similar to amphiphilic homopolymers [30,31], this value of *N* is about the minimal chain length at which the formation of the vesicle is possible.

For the longer chains, vesicles can be formed at weaker electrostatic interactions, and the area of the vesicles (and perforated vesicles) on the diagram extends for the poor solvent conditions (Figure 7b). The region of the hollow structures with the porous shell is very small for the short chains and significantly enlarges for the long chains. With the increase in chain length, the region of the layered, so-called onion-like structures (see Figure 5f), denoted on the diagram by half-shaded black circles, emerges.

**Figure 8:**
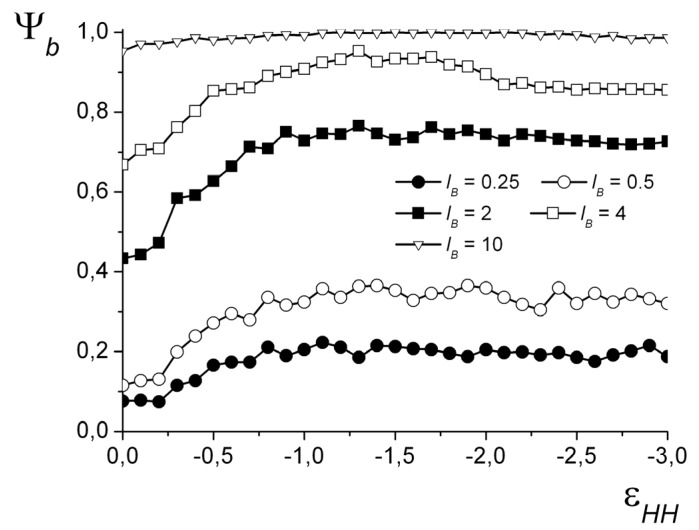
The fraction of groups forming ion pairs depending on $\varepsilon_{HH}$ at different *l_B_*. *N* = 1024.

For all the obtained morphologies, a fraction of the groups participating in the formation of the ion pairs was calculated: ψ*_b_* = *n_b_*/*N* (*n_b_* is the number of ion pairs). The oppositely charged groups are considered to form an ion pair when the distance *r* between them does not exceed a certain value *r_b_*. The chosen critical value is 1.5 of bond length (*r_b_* = 1.5*b*), which, assuming a typical monomer length of about 0.3 nm, corresponds to ~0.5 nm—a reasonable minimum charge-to-charge distance in IPECs. The ψ*_b_*($\varepsilon_{HH}$) dependencies are shown in Figure 8 for *N* = 1024. The shape of the curves at each value of *l_B_* does not significantly depend on *N*. For small *l_B_*, the value of ψ*_b_* grows with the decrease in the solvent quality and then reaches a plateau. That corresponds to the spherical core–shell, cylindrical, and toroid morphologies that have moderate ψ*_b_* values. For the spherical structures “dense hydrophobic core–loose hydrophilic shell,” ψ*_b_* does not exceed 0.25, and for cylindrical morphologies, it does not exceed 0.4. The stronger the electrostatic interactions, the higher the curve is located. For the strong electrostatic interactions (*l_B_* ≥ 4), the curves are located more densely with the increase in *l_B_*. For *l_B_* = 4, the value of ψ*_b_* grows, reaches the maximum of ψ*_b_* ≈ 0.95, and then decreases to the plateau of ψ*_b_* ≈ 0.85. The highest values of ψ*_b_* are achieved when the complex has either a vesicle-like or the layered structure. The slight decrease in ψ*_b_* is related to the deformation of the vesicle shell and the subsequent transfer of some hydrophilic groups into the volume of the cavity.

## 3. Analytical Theory

### 3.1. Model

In the framework of the analytical theory, all the conformational states of the IPEC obtained by computer simulation can hardly be described using only one approach. Therefore, we focus on the possibility of vesicle formation and a “spherical core–shell”—“vesicle” morphological transition.

As in the computer simulation study, we consider a stoichiometric complex comprising two macromolecules of the same length *N* (*N* >> 1) and ionization degree *f* in a selective solvent, which is very good for the polyanion and extremely poor for the polycation. Ionized monomer units are uniformly distributed along the polymer chains and carry (in absolute value) elementary charge *q*. Let $\upsilon_{P}$ be the volume and $a_{P}$ be the size of a monomer unit of a hydrophilic polyanion, and $\upsilon_{S}$ be the volume of solvent molecules. The volume and size of the monomer units of hydrophobic polycations are denoted by $\upsilon_{H}$ and $a_{H}$, respectively. Here, $\upsilon_{P}$ = $\upsilon_{H}$ = υ, $a_{P}$ = $a_{H}$ = a.

**Figure 9:**
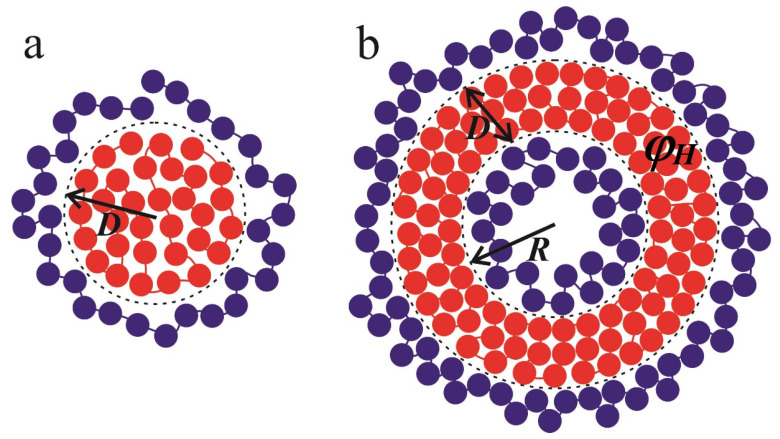
The schematic representation of spherical core–shell (**a**) and vesicular structures (**b**) considered in the framework of the analytical theory. The red circles depict monomer units of the polycations; the polyanions are shown by blue circles. The dashed lines represent the boundaries of the hydrophobic region. The radius of the spherical hydrophobic core is denoted by *D*, whereas the radius of the cavity equals *R* and the thickness of the spherical layer is *D*.

The strong short-range attraction between the monomer units of the polycation causes the formation of a dense globule (not necessary of a spherical shape) with a constant polymer concentration and a uniform distribution of charged groups. Meanwhile, the hydrophilic polymer chain is adsorbed onto the surface of the globule due to electrostatic interactions (Figure 9). 

The total free energy is expressed as a sum of six terms:

(9) $$F=F_{vol}+W+F_{surf}+F_{el-st}+F_{exc}+F_{conf}$$

where $F_{vol}$ is the free energy of the short-range interactions in the volume of the globule, W is the electrostatic energy of the globule, $F_{surf}$ is the surface free energy of the globule, $F_{el-st}$ describes electrostatic interactions of oppositely charged macromolecules, $F_{exc}$ accounts for excluded volume interactions of polyanion monomer units, and $F_{conf}$ is the conformational energy of polyanion.

The first term is written in the framework of the Flory–Huggins model and describes non-electrostatic interactions in the globule (here and below $\beta =\frac{1}{k_{B}T}$):

(10) $$\beta F_{vol}=N\chi \left(1-\varphi_{H}\right)+n_{S}\ln\left(1-\varphi_{H}\right)$$

Here, $\varphi_{H}$ is the volume fraction of hydrophobic groups; $n_{s}$ is the number of solvent molecules in the globule: $n_{s}=N\frac{\upsilon_{H}}{\upsilon_{S}}\frac{1-\varphi_{H}}{\varphi_{H}}$, and *χ* is a Flory–Huggins parameter of pair interactions between the polycation and the solvent molecules.

The electrostatic self-energy of the globule can be represented in terms of the electric field in the form:

(11) $$W=\frac{\varepsilon \varepsilon_{0}}{2}\int E^{2}dV$$

where *E* is the electric field in the globule; and ε and ε_0_ are the medium permittivity and the vacuum permittivity, respectively. The integration in Equation (11) is performed over the volume of the globule.

We consider the case of a very poor solvent quality (*χ* >> 1) for polycations, so almost all solvent molecules leave the globule, the volume fraction of polycation is close to unity, $\varphi_{H}$ ≈ 1, and the thickness of the surface boundary layer, *δ*, is of the order of monomer size, *δ* ~$a_{H}$. In this case, the conformational entropy of the chain makes a minor contribution to the surface energy as compared to the polymer–solvent interactions at the interface. As the surface free energy is defined as a difference between the energy of monomer units in the *δ* surface layer and inside the globule, one can estimate the surface free energy in the following form (see Appendix in [32]):

(12) $$\beta F_{surf}=\frac{a_{H}}{\upsilon_{H}}\chi \varphi_{H}^{2}S$$

where S is the total surface area of the globule.

The energy of excluded volume interactions between the monomer units of a soluble polyanion, described by the second virial coefficient B, is

(13) $$\beta F_{exc}=\int \frac{1}{2}Bc_{P}^{2}dV$$

wherein the concentration $c_{P}$ of monomer units of the polyanion near the globule is assumed to be quite small.

The electrostatic energy of the interaction between the adsorbed polyanion and the globule formed by the polycation can be expressed as [62]

(14) $$F_{el-st}=\int \left(-\frac{\varepsilon \varepsilon_{0}}{2}{\left(\vec{\nabla }\psi \right)}^{2}+\rho_{P}\psi \right)dV$$

where *ψ* is an electrostatic potential outside the globule; the charge density around the globule is related to the concentration of polyanions: $\rho_{P}=-qfc_{P}$. The first term in the parentheses of Equation (14) corresponds to the self-energy of the electric field, whereas the second one is the electrostatic energy of the ionized groups of the macromolecules.

The conformational loss due to the inhomogeneous distribution of monomer units of the polyanion is calculated as [13,63,64]

(15) $$\beta F_{conf}=\frac{a_{P}^{2}}{6}\int \frac{{\left(\vec{\nabla }c_{P}\right)}^{2}}{c_{P}}dV$$

In the present theory, it is assumed that $B>>\upsilon_{P}$, and the fraction of the charged groups in the chains is high (*f* ~ 0.5). Under these conditions, the electrostatic attraction (Equation (14)) between the polyanion and oppositely charged globule is stabilized by the excluded volume interactions (Equation (13)) between the monomers of the polyanion. Let us estimate the electrostatic energy $F_{el-st}$ in the case of the spherical core–shell structure. The electrostatic interaction energy is proportional to $\frac{N^{2}}{D}$ and the size of the globule $D\sim N^{\frac{1}{3}}$, whereas $F_{conf}$ exhibits a linear dependence on *N*. Then, the terms (Equation (13)) and (Equation (14)) are much greater than the conformational free energy (Equation (15)) if $\frac{N^{2}}{D}\sim N^{\frac{5}{3}}>>N$. Thus, the term (Equation (15)) can be neglected at N>>1 when the total free energy (Equation (9)) is varied with respect to the concentration of polyanions, $c_{P}$ (see Appendix A).

#### 3.1.1. The “Core–Shell” Structure

Firstly, let us consider the case when a polycation forms the spherical globule (Figure 9a), while the polyanion adsorbs onto it due to electrostatic interactions. The complex has a “core–shell” structure. The radius of the spherical globule is D; its volume and surface area are equal to $V_{C-S}=\frac{4\pi }{3}D^{3}$ and $S_{C-S}=4\pi D^{2}$, respectively. The volume fraction of uniformly distributed monomers in the globule is $\varphi_{H}=N\upsilon_{H}/V_{C-S}$.

The sum of electrostatic and excluded volume interactions can be found in Appendix A (A21):

(16) $$\beta F_{el-st}+\beta F_{exc}=-\frac{1}{2}\frac{l_{B}f^{2}N^{2}}{D\left(1+\kappa D\right)}$$

where *κ* is the inverse screening, $\kappa =\sqrt{\frac{4\pi f^{2}l_{B}}{B}}$; $l_{B}=q^{2}/\left(4\pi \varepsilon \varepsilon_{0}k_{B}T\right)$ is the Bjerrum length.

The electrostatic self-energy of the globule per monomer unit equals (see Appendix B, expression (A25))

(17) $$\beta W=\frac{1}{10}\frac{l_{B}f^{2}N^{2}}{D}$$

Thus, the total free energy per monomer unit for the “core–shell” structure is

(18) $$\beta F_{C-S}=N\chi \left(1-\varphi_{H}\right)+N\frac{\upsilon_{H}}{\upsilon_{S}}\frac{1-\varphi_{H}}{\varphi_{H}}\ln\left(1-\varphi_{H}\right)+\frac{1}{10}\frac{l_{B}f^{2}N^{2}}{D}+\frac{4\pi D^{2}a_{H}}{\upsilon_{H}}\chi \varphi_{H}^{2}-\frac{1}{2}\frac{l_{B}f^{2}N^{2}}{D\left(1+\kappa D\right)}$$

The free energy (18) for the core–shell structure is minimized with respect to the globule radius, *D*.

#### 3.1.2. Vesicle

Now, let us turn to the vesicular structure. The inner radius of the vesicle is denoted as $R_{in}=R$, and the external radius is $R_{out}=R+D$, where *D* is the thickness of the spherical layer (Figure 9b). Then, the inner and outer surface areas are equal to $S_{in}=4\pi R^{2}$ and $S_{out}=4\pi {\left(R+D\right)}^{2}$, while the volume of the spherical layer is defined by $V_{ves}=\frac{4\pi }{3}\left({\left(R+D\right)}^{3}-R^{3}\right)$. The volume fraction of monomer units of the polycation is given by $\varphi_{H}=N\upsilon_{H}/V_{ves}$.

The sum of electrostatic and excluded volume interactions for the inner and outer surfaces of the vesicle is derived in Appendix A (Equation (A20)):

(19) $$\begin{array}{l} \beta F_{el-st}+\beta F_{exc}=-\frac{1}{2}\frac{l_{B}f^{2}N^{2}{\left(1-y\right)}^{2}}{\left(R+D\right)\left(1+\kappa \left(R+D\right)\right)}-\\ -\frac{\kappa l_{B}f^{2}N^{2}y^{2}}{4{\left(\kappa R\cosh\left(\kappa R\right)-\sinh\left(\kappa R\right)\right)}^{2}}\left(\sinh\left(2\kappa R\right)-\frac{1}{\kappa R}\left(\cosh\left(2\kappa R\right)-1\right)\right) \end{array}$$

where the fraction y of monomer units of the polycation in the inner surface layer of thickness $a_{H}$ is $y=\varphi_{H}S_{in}a_{H}/\left(N\upsilon_{H}\right)$.

The electrostatic self-energy of this structure per monomer unit is calculated in Appendix B (Equation (A24)):

(20) $$\beta W=\frac{1}{2}\frac{l_{B}f^{2}N^{2}{\left(1-y\right)}^{2}}{{\left(3R^{2}D+3RD^{2}+D^{3}\right)}^{2}}\left(\frac{D^{3}R^{3}}{R+D}+2D^{3}R^{2}+D^{4}R+\frac{D^{5}}{5}\right)$$

The total free energy of the vesicle is

(21) $$\begin{array}{l} \beta F_{ves}=N\chi \left(1-\varphi_{H}\right)+N\frac{\upsilon_{H}}{\upsilon_{S}}\frac{1-\varphi_{H}}{\varphi_{H}}\ln\left(1-\varphi_{H}\right)+\\ +\frac{1}{2}\frac{l_{B}f^{2}N^{2}{\left(1-y\right)}^{2}}{{\left(3R^{2}D+3RD^{2}+D^{3}\right)}^{2}}\left(\frac{D^{3}R^{3}}{R+D}+2D^{3}R^{2}+D^{4}R+\frac{D^{5}}{5}\right)+\\ +\frac{4\pi \left({\left(R+D\right)}^{2}+D^{2}\right)a_{H}}{\upsilon_{H}}\chi \varphi_{H}^{2}-\frac{1}{2}\frac{l_{B}f^{2}N^{2}{\left(1-y\right)}^{2}}{\left(R+D\right)\left(1+\kappa \left(R+D\right)\right)}-\\ -\frac{\kappa l_{B}f^{2}N^{2}y^{2}}{4{\left(\kappa R\cosh\left(\kappa R\right)-\sinh\left(\kappa R\right)\right)}^{2}}\left(\sinh\left(2\kappa R\right)-\frac{1}{\kappa R}\left(\cosh\left(2\kappa R\right)-1\right)\right) \end{array}$$

Minimization of the free energy of the vesicle (Equation (21)) is performed with respect to the cavity radius *R* and its thickness *D*.

### 3.2. Analytical Results

To find the regions of stability of the considered structures, one should minimize the free energies $F_{C-S}$ (Equation (18)) and $F_{ves}$ (Equation (21)) and compare their equilibrium values for various parameters.

**Figure 10:**
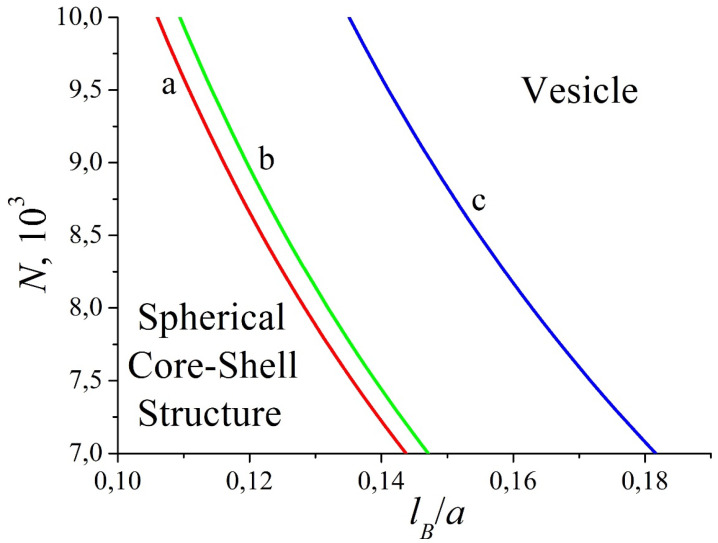
The morphological diagram of the complex in the coordinates “polymerization degree–Bjerrum length” for different degrees of ionization and second virial coefficients: *f* = 0.5, $B/\upsilon_{H}$ = 3 (**a**); *f* = 0.5, $B/\upsilon_{H}$ = 4 (**b**); *f* = 0.45, $B/\upsilon_{H}$ = 4 (**c**). Other parameters: $\upsilon_{H}/\upsilon_{S}$ = 1, *χ* = 6, $\upsilon_{H}/a_{H}^{3}=1$.

The structure of the complex is mainly defined by the balance between the electrostatic and short-range hydrophobic interactions. For sufficiently small values of *N* and *l_B_*, the formation of a core–shell structure is favorable, as the surface energy (hydrophobic interactions) and excluded volume interactions of hydrophilic macromolecules play the main role. However, if one increases the degree of polymerization or/and the Bjerrum length, the attraction of oppositely charged macromolecules becomes stronger and the complex transformation into the spherical layer is accompanied by the growth of the surface area of the hydrophobic core. The vesicle cavity diameter, 2*R*, is greater than the monomer size of the polyanion, 2*R* ≈ $10a_{P}$ at the transition line and grows with the further increase in the parameters *N* and *l_B_*. The decrease in the degree of ionization is related to the lowering of electrostatic interactions, which results in an increase in the core–shell region in the diagram (cf. Figure 10, curves b and c). When the second virial coefficient of polycation–solvent interactions increases, the transition line shifts toward higher *N* and *l_B_* (cf. Figure 10, curves a and b). In addition, the excluded volume interactions dominate and the hydrophilic polymer chain becomes more swollen. In this case, vesicle formation is possible if electrostatic interactions become stronger (larger *N* and *l_B_*), as part of the polyanions adsorbed onto the inner surface of the core is trapped in the cavity and, therefore, the excluded volume free energy is high. 

In the theoretical consideration, we only investigate the possible formation of morphologies having spherical symmetry. In fact, in computer simulations, the transition from a spherical core–shell structure to a non-spherical one (cylinder, torus), rather than directly to the vesicle, happens (Figure 7b, dashed lines). Nevertheless, the present analytical theory gives insights into physical reasons for such a transition and provides favorable parameters for the self-assembly of the spherical layer. The region of stability of the spherical core–shell structures is located at the lower left corner of the diagrams, whereas the vesicle region corresponds to the higher values of *N* and *l_B_* and enlarges with the increase in *N*, which is in perfect agreement with the analytical results (cf. Figure 7b and Figure 10). It should be stressed that, in theory, we suppose large values of the second virial coefficient ($B/\upsilon_{P}>>1$) to obtain a solution of the Poisson equation in a closed form. In addition, the calculations show that higher *B* values are related to longer macromolecules at the transition point when the other parameters are fixed. However, the computer simulation deals with the moderate values of *B* ($B\sim \upsilon_{P}$) determined by the Lennard–Jones potential (Equation (2)). For example, at *N* = 512, the vesicle is formed with a very small cavity that is almost completely filled by polyanion monomer units. This situation is opposite to the theoretical assumption of low concentrations of the adsorbed macroion. Therefore, the choice of relatively high *N* compared to the computer simulation is dictated by original assumptions of large values of the second virial coefficient and a small concentration of the adsorbed polyanion.

## 4. Conclusions

In the present study, by means of computer simulation, morphological diagrams of the interpolyelectrolyte complexes containing polyions with different affinities for the solvent were built. The morphologies taken by the complex in dilute solution depend on the solvent quality for one of the polyions, the length of the chains, and the Bjerrum length. Formation of the various morphologies is due to both the hydrophobic and electrostatic interactions. Groups of the polyions with hydrophobic units tend to be surrounded mostly by groups of the same chain, and the hydrophilic groups tend to be exposed into the solvent. The electrostatic interactions play the role of the “bonds” holding the whole complex together. Therefore, at sufficiently strong electrostatic interactions, there is a range of the parameter $\varepsilon_{HH}$, related to the hydrophobic interactions between the units of one of the polyions, where the complex can take the shape of the hollow particle. 

Along with the vesicle-like structures with the continuous walls of uniform thickness, perforated vesicles with the symmetric distribution of the pores were observed. The number of the pores decreases and the type of symmetry changes with the worsening of solvent quality. Such perforated vesicles transform into solid structures with strongly segregated symmetrically arranged chains.

An approach to the estimation of the pore distribution in the perforated vesicles was proposed. It is based on the spherical harmonics expansion of the vesicle wall thickness.

The analytical theory was applied to study the vesicle structure and the core–shell-to-vesicle transition. It was shown that the shorter macromolecules form a core–shell structure, while the appearance of vesicles is preferable at sufficiently large degrees of polymerization and Bjerrum length that can correspond to low values of medium permittivity. The increase in the degree of ionization also promotes vesicle formation. The theoretical results corroborated that with the increase in the electrostatic interactions and the length of the macroions, the hollow morphologies are more favorable than the core–shell ones.

## Appendix A

To find the equilibrium value of the free energy (Equation (9)) at a fixed volume fraction of polycation $\varphi_{H}$, one should minimize the following function with respect to the concentration of polyanion $c_{P}\left(r\right)$ and the electrostatic potential ψ(r):

(A1) $$\beta F\left(c\left(r\right),\psi \left(r\right)\right)=const+\int \left(-\beta \frac{\varepsilon \varepsilon_{0}}{2}{\left(\vec{\nabla }\psi \right)}^{2}+\beta \rho_{P}\psi +\frac{1}{2}Bc_{P}^{2}+\frac{a_{P}^{2}}{6}\frac{{\left(\vec{\nabla }c_{P}\right)}^{2}}{c_{P}}\right)dV$$

As discussed in the main text, in the present theoretical consideration, we neglect the last term in Equation (A1). The variation in the free energy with respect to $c_{P}(r)$ gives us the relationship between the concentration of the adsorbed polymer chain $c_{P}$ and electrostatic potential ψ(r):

(A2) $$c_{P}(r)=\frac{\beta qf\psi (r)}{B}$$

The charge density around the globule equals (“minus” sign is related to negatively charged groups)

(A3) $$\rho_{P}(r)=-qfc_{P}(r)=-\frac{\beta q^{2}f^{2}\psi (r)}{B}$$

The variation in the free energy (A1) with respect to ψ gives the Poisson equation:

(A4) $$\Delta \psi =-\frac{\rho_{P}}{\varepsilon \varepsilon_{0}}$$

Substituting (A3) in (A4), we finally obtain

(A5) $$\Delta \psi =\kappa^{2}\psi$$

Here, we introduce the inverse screening radius $\kappa =\sqrt{\frac{4\pi f^{2}l_{B}}{B}}$; $l_{B}=q^{2}/(4\pi \varepsilon \varepsilon_{0}k_{B}T)$ is the Bjerrum length. The inverse screening radius is the ratio of magnitudes of the electrostatic and excluded volume interactions. The higher the second virial coefficient B, the less effective the screening.

It is worth noting that the constraint

(A6) $$\int c_{P}dV=N,$$

holds automatically. Indeed, integrating Equation (A4) and using boundary conditions (see below), one can prove that the volume charge near the surface of the globule, formed by hydrophobic polycations, is equal to the amount of charge that creates the electrostatic field $E_{0}$ at the surface of the globule. Hence, the total charge of the complex is zero, from which it follows that the equality (A6) holds.

Let us introduce the following integrals:

(A7) $$I_{1}=-\beta {\int \frac{\varepsilon \varepsilon_{0}}{2}\left(\vec{\nabla }\psi \right)}^{2}dV,$$

(A8) $$I_{2}=\beta \int \rho_{P}\psi dV$$

Then,

(A9) $$\beta F_{el-st}=I_{1}+I_{2}$$

Using (A2), one can show that

(A10) $$I_{2}=\int \rho_{P}\psi dV=-2F_{exc}.$$

Therefore, the sum of electrostatic and excluded volume interactions is equal to

(A11) $$\beta F_{el-st}+\beta F_{exc}=I_{1}+\frac{1}{2}I_{2}$$

The fraction of monomer units on the inner surface (its area is $S_{in}=4\pi R^{2}$) of the globule in the layer of thickness $a_{H}$ is $y=S_{in}a_{H}\varphi_{H}/(N\upsilon_{H})=3R^{2}a_{H}/\left(3R^{2}D+3RD^{2}+D^{3}\right)$. The number of monomer units on the inner surface can be estimated as $N_{in}=Ny$. Then, the amount of charge on the inner surface is $Q_{in}=qfN_{in}=qfNy$. The remaining charge $Q_{out}=qfN-Q_{in}=qfN(1-y)$ is assumed to be uniformly distributed in the volume of the spherical layer.

The vesicle has inner and outer surfaces, so one should, therefore, solve corresponding interior and exterior boundary problems in the case of spherical geometry.

First, we consider the exterior boundary problem for the Poisson equation:

(A12) $$\left\{ \begin{array}{l} \frac{d^{2}\psi_{out}}{dr^{2}}+\frac{2}{r}\frac{d\psi_{out}}{dr}=\kappa^{2}\psi_{out}\\ \psi_{out}(\infty )=0\\ {-\frac{d\psi_{out}(r)}{dr}|}_{r=R_{out}}=E_{out} \end{array}\right.$$

The electrostatic field at the outer surface of the vesicle is $E_{out}=\frac{Q_{out}}{4\pi \varepsilon \varepsilon_{0}R_{out}^{2}}$. Using the first condition, we can conclude that the solution of the equation is a decaying function: $\psi_{out}(r)=C\frac{e^{-\kappa r}}{r}$. From the second boundary condition, we obtain the constant C:

(A13) $$\psi_{out}(r)=\frac{qfN(1-y)}{4\pi \varepsilon \varepsilon_{0}(1+\kappa R_{out})}\frac{e^{\kappa (R_{out}-r)}}{r}$$

The evaluation of the integrals (A7) and (A8):

(A14) $$I_{1}=-\beta \int_{R_{out}}^{\infty }\frac{\varepsilon \varepsilon_{0}}{2}{\left(\vec{\nabla }\psi \right)}^{2}dV=-\frac{1}{2}\frac{\kappa l_{B}f^{2}N^{2}{\left(1-y\right)}^{2}}{{\left(1+\kappa R_{out}\right)}^{2}}\left(\frac{1}{2}+\frac{1}{\kappa R_{out}}\right),$$

(A15) $$I_{2}=\beta \int_{R_{out}}^{\infty }\rho_{P}\psi dV=-\frac{1}{2}\frac{\kappa l_{B}f^{2}N^{2}{\left(1-y\right)}^{2}}{{\left(1+\kappa R_{out}\right)}^{2}}.$$

In the case of the interior boundary problem, we have:

(A16) $$\left\{ \begin{array}{l} \frac{d^{2}\psi_{in}}{dr^{2}}+\frac{2}{r}\frac{d\psi_{in}}{dr}=\kappa^{2}\psi_{in}\\ \left|\psi_{in}(0)\right|<\infty \\ {\frac{d\psi_{in}(r)}{dr}|}_{r=R_{in}}=E_{in} \end{array}\right.$$

Here, $E_{in}=\frac{Q_{in}}{4\pi \varepsilon \varepsilon_{0}R_{in}^{2}}$ is the electrostatic field at the outer surface of the vesicle. The condition $\left|\psi_{in}(0)\right|<\infty$ gives a solution of the equation in the form: $\psi_{in}(r)=C\frac{\sinh(\kappa r)}{r}$.

Note that there is no “minus” sign in front of ${\frac{d\psi_{in}(r)}{dr}|}_{r=R_{in}}$, as $r-R_{in}\leq 0$ for an interior boundary problem (cf. (A12)). From the second boundary condition, we obtain the constant C:

(A17) $$\psi_{in}(r)=\frac{Q_{in}}{4\pi \varepsilon \varepsilon_{0}\left(\kappa R_{in}\cosh(\kappa R_{in})-\sinh(\kappa R_{in})\right)}\frac{\sinh(\kappa r)}{r}.$$

One can evaluate integrals (A6) and (A7):

(A18) $$\begin{array}{l} I_{1}=-\beta \int_{0}^{R_{in}}\frac{\varepsilon \varepsilon_{0}}{2}{\left(\vec{\nabla }\psi_{in}\right)}^{2}dV=-\frac{\kappa l_{B}f^{2}N^{2}y^{2}}{2{\left(\kappa R_{in}\cosh(\kappa R_{in})-\sinh(\kappa R_{in})\right)}^{2}}.,\\ .\left(\frac{1}{2}\kappa R_{in}+\frac{1}{4}\sinh(2\kappa R_{in})-\frac{1}{2\kappa R_{in}}\cosh(2\kappa R_{in})+\frac{1}{2\kappa R_{in}}\right) \end{array}$$

and

(A19) $$I_{2}=\beta \int_{0}^{R_{in}}\rho_{P}\psi_{in}dV=-\frac{\kappa l_{B}f^{2}N^{2}y^{2}}{{\left(\kappa R_{in}\cosh\left(\kappa R_{in}\right)-\sinh\left(\kappa R_{in}\right)\right)}^{2}}\left(\frac{1}{4}\sinh\left(2\kappa R_{in}\right)-\frac{1}{2}\kappa R_{in}\right).$$

Finally, we obtain the sum of excluded volume and electrostatic free energies:

(A20) $$\begin{array}{l} \beta F_{exc}+\beta F_{el-st}=-\frac{1}{2}\frac{l_{B}f^{2}N^{2}{\left(1-y\right)}^{2}}{\left(R+D\right)\left(1+\kappa \left(R+D\right)\right)}-\\ -\frac{\kappa l_{B}f^{2}N^{2}y^{2}}{4{\left(\kappa R\cosh\left(\kappa R\right)-\sinh\left(\kappa R\right)\right)}^{2}}\left(\sinh\left(2\kappa R\right)-\frac{1}{\kappa R}\left(\cosh\left(2\kappa R\right)-1\right)\right) \end{array}.$$

When there is no cavity (R=0), we recover $F_{exc}+F_{el-st}$ for the spherical core–shell structure:

(A21) $$\beta F_{exc}+\beta F_{el-st}=-\frac{1}{2}\frac{l_{B}f^{2}N^{2}{\left(1-y\right)}^{2}}{D\left(1+\kappa D\right)}.$$

## Appendix B

The charge density in the spherical layer is equal to $\rho_{H}=\frac{fN(1-y)}{\frac{4\pi }{3}(3R^{2}D+3RD^{2}+D^{3})}$. The electric field E(r) in the globule volume can be found from Gauss’s law:

(A22) $$E(r)=\frac{\rho_{H}}{3\varepsilon \varepsilon_{0}}(r-\frac{R^{3}}{r^{2}}).$$

In particular, the electrostatic field at the outer surface is equal to

(A23) $$E(R_{out})=\frac{Q_{out}}{4\pi \varepsilon \varepsilon_{0}R_{out}^{2}}=E_{out},$$

which gives a boundary condition for the exterior boundary problem (A12).

Then, the electrostatic self-energy of the spherical layer is equal to

(A24) $$\beta W=\beta \frac{\varepsilon \varepsilon_{0}}{2}\int_{R}^{R+D}4\pi r^{2}E^{2}dr=\frac{1}{2}\frac{l_{B}f^{2}N^{2}{\left(1-y\right)}^{2}}{{\left(3R^{2}D+3RD^{2}+D^{3}\right)}^{2}}(\frac{D^{3}R^{3}}{R+D}+2D^{3}R^{2}+D^{4}R+\frac{D^{5}}{5}).$$

To obtain the similar electrostatic energy for the spherical globule, one should set the radius of the vesicle cavity to zero, R=0. Thus, the electrostatic self-energy becomes

(A25) $$\beta W=\beta \frac{\varepsilon \varepsilon_{0}}{2}\int_{0}^{D}4\pi r^{2}E^{2}dr=\frac{1}{10}\frac{l_{B}f^{2}N^{2}}{D}.$$
